# Process-Oriented Profiling of Speech Sound Disorders

**DOI:** 10.3390/children9101502

**Published:** 2022-09-30

**Authors:** Sanne Diepeveen, Hayo Terband, Leenke van Haaften, Anne Marie van de Zande, Charlotte Megens-Huigh, Bert de Swart, Ben Maassen

**Affiliations:** 1 HAN University of Applied Sciences, 6524 TM Nijmegen, The Netherlands; 2 Donders Institute for Brain, Cognition and Behaviour, Department of Rehabilitation, Radboud University Medical Center, 6525 AJ Nijmegen, The Netherlands; 3 Department of Communication Sciences and Disorders, University of Iowa, Iowa City, IA 52242, USA; 4 Rijndam Rehabilitation Centre Rotterdam, 3001 KD Rotterdam, The Netherlands; 5 Centre for Language and Cognition, Groningen University, 9712 EK Groningen, The Netherlands

**Keywords:** speech sound disorders, diagnosis, treatment planning

## Abstract

The differentiation between subtypes of speech sound disorder (SSD) and the involvement of possible underlying deficits is part of ongoing research and debate. The present study adopted a data-driven approach and aimed to identify and describe deficits and subgroups within a sample of 150 four to seven-year-old Dutch children with SSD. Data collection comprised a broad test battery including the Computer Articulation Instrument (CAI). Its tasks Picture Naming (PN), NonWord Imitation (NWI), Word and NonWord Repetition (WR; NWR) and Maximum Repetition Rate (MRR) each render a variety of parameters (e.g., percentage of consonants correct) that together provide a profile of strengths and weaknesses of different processes involved in speech production. Principal Component Analysis on the CAI parameters revealed three speech domains: (1) all PN parameters plus three parameters of NWI; (2) the remaining parameters of NWI plus WR and NWR; (3) MRR. A subsequent cluster analysis revealed three subgroups, which differed significantly on intelligibility, receptive vocabulary, and auditory discrimination but not on age, gender and SLPs diagnosis. The clusters could be typified as three specific profiles: (1) phonological deficit; (2) phonological deficit with motoric deficit; (3) severe phonological and motoric deficit. These results indicate that there are different profiles of SSD, which cover a spectrum of degrees of involvement of different underlying problems.

## 1. Introduction

A substantial part of the caseload of speech and language pathologists (SLPs) consists of children with a speech sound disorder (SSD). Prevalence estimates vary, ranging from approximately 3.4% to 24.6% of children in the age of 4 to 8 years being diagnosed with an SSD [1,2,3]. Children with SSD are a heterogenous group in terms of symptoms and severity as well as regarding (suspected) underlying deficits (and comorbidities), which makes diagnosing children with SSD a complicated affair [4,5].

### 1.1. Speech Development

Speech is the product of a variety of linguistic and speech motor processes working together [6,7,8,9]. During speech production, the first process is the conceptualization of a preverbal message from memory or from perception, for example seeing a picture of a cat in a naming task. Next is the formation of an utterance (word or sentence), which is executed by two lexicalization steps: the selection of a lemma, which contains meaning and grammatical word information, and the related lexeme or word form. This lexeme is the input for the next phase, phonological encoding, which consists of generating the sequence of speech sounds and the syllabic and prosodic structures. The selected syllables are the basic elements of the next phase: articulomotor planning and programming. Here, the motor plans and programs for the different speech movements are formed. Motor planning involves the selection and sequencing of articulatory movement goals which are then implemented in muscle specific motor programs (motor programming). Finally, the articulatory movements are executed (motor execution). The neural signals are sent to peripheral systems and transformed into coordinated muscle activity, resulting in an acoustic speech signal [10,11,12,13].

Children develop adult-like speech both through the development of motor skills and through the expansion of the language system, especially the storage of words with their associated phonemes (lexeme) and the sound system (phonology). Around the age of 24 months, an expressive vocabulary spurt is observed in typically developing (TD) children. During this spurt, a temporary increase in the variability of jaw movements is found, which is believed to be due to the speech motor system rearranging itself to match the rapid cognitive and linguistic development [14,15,16]. Saletta et al. [17] found that a task with a higher linguistic load was associated with increased speech motor variability in TD children’s speech. Thus, linguistic/phonological development influences the speech motor system and vice versa. Both developmental systems can present problems in children with SSD and in intervention an SLP should use different therapy methods for the two systems. An SLP has to investigate both systems in the diagnostic phase. Problems of interpretation arise when an SLP uses only a naming task in the assessment process. Naming the picture of, for example, a cat in a speech assessment does not provide enough information to differentiate a linguistic deficit from a speech motor deficit based on speech errors alone (overt symptoms). If, in the example of the target word ‘cat’, the /k/ is substituted into [t] this may be interpreted as the phonological process of fronting; the child substitutes a sound produced with the tongue further back in the mouth for one made with the tongue tip just behind the teeth, at the front of the mouth. However, this substitution can also be seen as a simplification of the word ‘cat’; the child uses not two different articulatory movement goals, /k/ and /t/, but only one which is easier to produce. The present study set out to investigate the results of a process-oriented speech assessment in a large sample of children with SSD. Using a data-driven approach we investigated if subgroups can be distinguished and how they compare.

### 1.2. Current Practice in Speech Assessments and Interpretation

As mentioned above, diagnosing children with SSD is a hard task due to the ambiguity of the diagnostic markers for SSD subtypes and the overlap of speech symptoms between the different diagnostic labels. According to SLPs’ reports, a wide variety of different speech assessments are used to diagnose children with SSD and often more than one assessment is used for a single child/per case [18,19,20,21,22,23,24]. The obtained assessment data are interpreted based on the SLP’s own clinical experience and not on the basis of a clearly formulated set of objectified criteria, as evidenced from data from the Netherlands [18] and the United Kingdom [18,19]. From questionnaires and interviews of a total of 170 SLPs in the Netherlands, Diepeveen et al. [18] found that there is no consensus on the terminology and there are many idiosyncrasies in diagnosis and treatment planning of SSDs. A reported 85 different diagnostic labels were used for children with SSD and the speech symptoms associated with these labels showed large overlap. Furthermore, the reports indicated that intervention methods were used for a variety of different diagnostic labels and methods incongruent with their described purpose. The Nuffield Dyspraxia Programme, for example, was also used with children who had been diagnosed with a phonological problem [18]. Overall, the study concluded that there is no consensus among SLPs in the Netherlands on the terminology and there are many idiosyncrasies in diagnosis and treatment planning of SSDs.

SLPs have different classification systems at their disposal that differentiate subtypes of speech disorders in children (see Waring and Knight [25] for an overview). Two of the systems that are commonly used are Shriberg’s Speech Disorders Classification System (SDCS) [26] and Dodd’s Model of Differential Diagnosis (MDD) [27,28]. These two systems have a different approach on classifying SSD. The SDCS is based on the behavioral phenotype of the child’s speech and etiological background, whereas the MDD is based on a descriptive-linguistic approach. SDCS and MDD have been subject of prevalence studies, which are shortly summarized below.

The SDCS is an organized framework to distinguish between several subtypes of SSD. It has four levels: etiological processes (distal causes), speech processes (proximal causes), clinical typology (behavioral phenotypes) and diagnostic markers (critical signs of phenotype). At the clinical typology level, three different types are described; each characterized by a specific set of disorders. The three main groups are: speech delay (SD), speech errors (SE) and motor speech disorder (MSD [29]). In a study of 97 children with SSD, Vick et al. [30] discovered two groups of children based on five speech tasks and also non-speech tasks. One group (76%) met the criteria of SD and a smaller group (10.3%) met the criteria of motor speech disorder—not otherwise specified (MSD-NOS). Differences between the groups were on atypical speech movements such as a higher variability in measures of articulatory kinematics and a poor performance on iambic lexical stress word imitation in the MSD-NOS group. To further examine the use of the SDCS for the motor speech disorder group and to estimate the prevalence of the types of motor speech disorders, Shriberg et al. [29] used a sample of 415 children with idiopathic speech delay. A conversational speech sample of each child was used to complete a narrow phonetic transcription, a prosody-voice coding, and an acoustic analysis. These were then entered into the SDCS analysis program and based on the outcomes of the three measures a child was classified in a group. The classification of MSD applied, was Speech Motor Delay, Childhood Dysarthria, Childhood Apraxia of Speech (CAS), and concurrent Childhood Dysarthria and CAS. The following results emerged: 82.2% of the children that met the SDCS criterion for SD at assessment had no MSD; 17.8% with SD met criteria of one of the subgroups of MSD. Of the latter group, 12% was classified has having a Speech Motor Delay; 3.4% met criteria for Childhood Dysarthria and 2.4% children were classified with CAS. None of the children were classified has having the combination Childhood Dysarthria and CAS.

Another model that is often used by SLPs is Dodd’s [27] Model for Differential Diagnosis (MDD). The MDD model contains the following diagnostic labels: (1) articulation disorder: substitutions or distortions of sound (e.g., lateral lisp); (2) phonological delay: speech error patterns typical of younger children; (3) consistent atypical phonological disorder: consistent error patterns of unusual non-developmental errors; (4) inconsistent phonological disorder: inconsistent error pattern of the same lexical item and no oromotor difficulties; and (5) CAS: inconsistency in speech, oromotor signs, slow speech rate, disturbed articulation, short utterance length, poorer performance in imitation. For each of these labels a description is given of the speech problems that can be seen during assessment (Dodd, 2014). Ttofari-Eecen et al. [28] conducted a validation study for the MDD model and assessed a group of children who speak standard Australian English with the Goldman-Fristoe Test of Articulation 2 (GFTA-2) (Sounds-in-Words and Stimulability sections [31]), the Diagnostic Evaluation of Articulation and Phonology (DEAP Inconsistency Assessment) [32] and the Verbal Motor Production Assessment for Children (VMPAC) [33]. A total of 126 children were eventually divided into the five groups: suspected atypical speech motor control (10%); inconsistent phonological disorder (15%); consistent atypical phonological disorder (20%); phonological delay (55%); and articulation disorder (0%). Ttofari-Eecen et al. [28] concluded that although the model was designed for the use of children with an articulation or phonological delay or disorder only, the model can be used by SLPs in clinical practice to differentiate children with suspected SSD including children with motor speech disorder such as dysarthria or CAS.

In the MDD and the SDCS, classification is done through the description of the error patterns of the speech output and these errors are compared with typically developing children. Within the SDCS, the extensive use of etiological criteria is also included [25]. The question is whether an SLP can differentiate between the different diagnostic labels based on the error pattern and/or etiology. Both the MDD and the SDCS models leave little room for selecting multiple diagnoses per child, as shown in the two studies described above; all 415 children in Shriberg et al. [29] and all 126 children in Ttofari-Eecen et al. [28] received only one diagnosis. Speech errors and/or the etiological background are matched to a specific diagnostic label in both models, and thus these classification systems seem to leave no room for diagnosing the gradual involvement of multiple underlying deficits belonging to one or more different diagnostic labels [9]. 

### 1.3. Diagnostic Profiling within the Psycholinguistic Framework

As mentioned above, some children with SSD present problems in multiple processes, both linguistic and speech motor [34]. An SLP should therefore assess these multiple processes in a child with SSD to find out which one or more of these underlying processes show deficient functioning. The Psycholinguistic Framework aids SLPs to examine at a cognitive or psycholinguistic level where in the speech and language process the impairment is situated [35]. This framework is a psycholinguistic speech-processing model and comprises a ‘box and arrow’ model of speech processing skills and representations that serves as a guide for compiling individual profiles of strengths and weaknesses [12]. By comparing speech symptoms under different elicitation conditions within this framework, the proximal causes of SSD can be studied since involvement of underlying processes is different in different speech conditions. In a nonword imitation setting, for example, an alternative speech production route starts from auditory input. Since the child has no lexical representation of the target nonword available, the child must use either the phonological decoding and encoding system (analyze and select combinations of familiar consonants and vowels, possibly syllables) or the auditory-to-motor-planning pathway (repeating the sounds without phonological interpretation, such as in repeating click-sounds). 

The problems experienced by children with SSD can be at the level of word-form retrieval, phonological encoding, motor planning and programming, and/or articulation (motor execution). Systematic comparison of speech symptoms under varying conditions allows for assessing a profile of intact and deficient processes. This calls for a shift in the clinical reasoning skills of SLPs from a more diagnostic classification system such as the MDD or the SDCS model (diagnostic categories based on error patterns within a naming task or spontaneous speech) to a process-orientated view [9]. In other words, an SLP should identify the possible deficiencies of the underlying speech processes [7,8,9]. Unfortunately, current diagnostic instruments are not designed to provide fine-grained information about the involvement of the different underlying speech production processes [9]. For example, Geronikou and Rees [36] conducted a small study to profile four Greek speaking children with SSD based on nonword auditory discrimination, mispronunciation detection, naming, real word repetition and nonword repetition. The children could be profiled as having issues with either phonological or motor representations and the authors concluded that there is a need for a study with a wider range of consonants and clusters in different positions in words in the diagnostic instrument and they also advised to use a larger group of children. Such a study is possible with a new diagnostic instrument developed and released in the Netherlands, the Computer Articulation Instrument (CAI) [37]. The basic idea of the CAI is that speech is elicited in different contexts, which each tap into different levels of the production process such that functioning of production processes can be assessed by comparing performances. In addition, the sample of elicited words and nonwords contain all consonants and clusters in different positions, in most cases in at least two different words/nonwords, depending on the frequency of occurrence of the consonants and clusters in the Dutch language. Thus, the instrument yields comprehensive speech profiles from several speech tasks that reflect the functioning of different speech production processes—including phonological skills and speech motor skills; a comparison of those speech profiles gives an indication of possible underlying deficits.

The first aim of the present study was to determine which components emerge in a sample of 150 children with SSD with Principal Component Analysis (PCA) of the speech measures (20 parameters) of the CAI. This analysis was previously conducted for a norm group of 1524 typically developing Dutch-speaking children aged between 2;0 and 7;0 (years;months) indicated five meaningful components: (1) picture naming (PN); (2) segmental quality of nonword imitation (NWI); (3) quality of syllabic structure of NWI; (4) word and nonword proportion of whole-word variability (PWV), based on word- and nonword repetition (WR and NWR); and (5) mono- and multi-syllabic sequences of maximum repetition rate (MRR) [13]. PCA is not premised on average skills, but on the variation of skills and particularly on covariance. In a typical population, variation of skills may not be expressed in specific underlying components, due to a ceiling effect. In contrast, in an SSD population, underlying deficits may cause large covariance. If the components are similar, this could mean that children with SSD go through similar developmental milestones as typically developing children, which could be interpreted as an overall speech delay. In contrast, a different component structure could imply a deviant speech profile, which would indicate specific speech deficits. The components can also provide information about the tasks in which specific speech symptoms appear, which helps interpretation regarding the psycholinguistic processes involved.

The second aim was to test whether profiles can be differentiated and identified with the CAI test battery [37] in the same sample of children with SSD. To this end, we conducted k-means cluster analysis, an unsupervised machine learning method to partition data into a *k* number of groups (clusters) by minimizing variances within clusters, maximizing group similarity. This analysis was exploratory with no preconceived hypotheses about how children would group.

## 2. Materials and Methods

### 2.1. Participants

Participants were recruited in collaboration with the SPEECH study [38]. 150 children aged 4;0 to 6;6 (years;months, M = 5;2) participated in this study. The sample consisted of 94 boys and 56 girls; this ratio between boys and girls is consistent with other international studies [2,29]. The children were recruited through private practices (*n* = 60), special schools for language- and hearing-impaired children (*n* = 60), a rehabilitation center (*n* = 16), regular schools (*n* = 12) and an audiological center (*n* = 2) in the Netherlands. The children lived in different regions of the Netherlands (North, *n* = 13; East, *n* = 44; South, *n* = 20; West, *n* = 73). Three children also spoke a language other than Dutch: German, English, and Spanish.

Inclusion criteria were as follows:

- Aged 4;0 to 6;11 (years;monthts);
- Dutch as the primary language as indicated by parental report;
- No history of hearing problems based on parents’ or caregivers’ information (further indicated by care givers) about the child’s hearing status;
- A speech sound disorder (SSD) diagnosed by the referring SLP.

At the time of the study 138 children received speech and language therapy. One of these children had scores on the CAI above percentile 16 (see below) and was excluded for this study. Twelve children were recruited through regular schools and had no history of speech or language therapy; they were recruited for the control group of another study [38] and were found to have an SSD. These cases were referred to an SLP and were added to the SSD group.

Diagnoses were based on clinical observation and/or a Dutch speech assessment (note that no normalized and standardized assessments were available at the time) and determined by the child’s SLP. The majority of the children were diagnosed with a phonological disorder (*n* = 105), seventeen children with CAS, nine children with a phonetic articulation disorder, five children with dysarthria and the diagnosis of two children was not further specified by the SLP. Eleven children (those recruited through regular schools and not receiving speech therapy at the time of the study) were not previously diagnosed and were referred to an SLP after the diagnostic session; these children did not receive a diagnostic label. Of all children, thirty-two children received more than one diagnosis; sixteen children were diagnosed with a phonological disorder in combination with a phonetic disorder; ten children with CAS and a phonological disorder; two children with dysarthria and a phonological disorder; one child with CAS and a phonetic disorder. Three children received three diagnoses (CAS, phonological disorder, and a phonetic disorder).

Receptive vocabulary of 123 children was determined with the Peabody Picture Vocabulary Test-III-NL [39] (*n* = 79) or another comprehension test (*n* = 44) was available in the child’s file. Ninety-one children had a quotient score above 85 (range 85–129; 32 children had a score below the 85 (range 66–84). The other children (*n* = 26) were judged to have a normal comprehension level of the Dutch language, as determined by a professional (teacher, daycare employee and/or SLP), caregivers and the examiner. Comprehension language scores within normal range were not an inclusion criterion, since a comorbidity of a language impairment is common for children with SSD [1].

### 2.2. Data Collection

Caregivers were first asked to complete a questionnaire containing questions about their child’s speech and language development, and health condition. They also completed the Intelligibility in Context Scale (ICS [40]). If the child already received speech therapy, the SLP was also asked to fill out a questionnaire about the child’s speech and language abilities. The children were subsequently seen during one or two sessions by 12 student-SLPs or SLPs specifically trained in the administration of the different assessments. The assessment took place at school, private practice, rehabilitation center, or audiological center facilities, in a quiet room.

### 2.3. Materials

**Figure 1:**
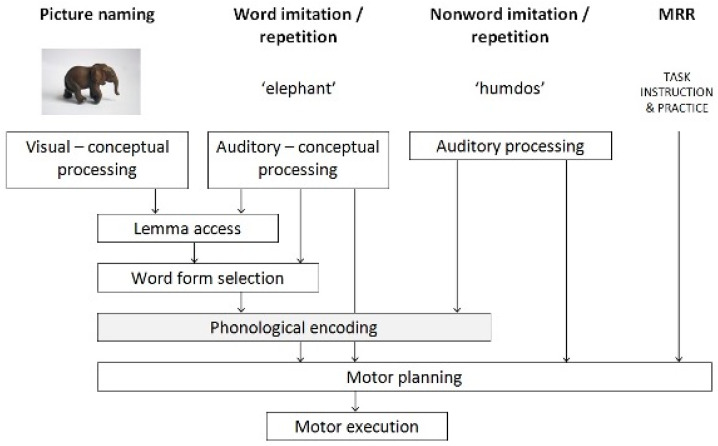
The speech production processes assessed in the four tasks of the Computer Articulation Instrument (Maassen & Terband [11]; Figure 15.2). MRR = maximum repetition rate. Printed with permission.

During the one or two sessions a receptive vocabulary task, the Peabody Picture Vocabulary Test-III-NL (PPVT-III-NL [39]); an auditory discrimination test (phonemic judgement) part of the Testinstrumentarium Taalontwikkelingsstoornissen (TTOS-ADT [41]) and the Computer Articulation Instrument (CAI) were conducted. The framework of the CAI is an integrated model of the cognitive and sensorimotor functions involved in speech production and perception (see Figure 1 [9,11]).

The first task of the CAI, picture naming (PN), examines the child’s ability to retrieve the stored information about a real word and contains the whole chain of the speech production process, from preverbal visual-conceptual processing to lemma access, word-form retrieval, phonological encoding, motor planning, and articulation (motor execution; see Figure 1 [11]). In the second task, the child is asked to imitate nonwords (NWI). Due to the nature of the task, the child has no lexical representation of the target utterance available, which means the child must use either the phonological decoding and encoding system or the auditory-to-motor-planning pathway. For the word (WR) and nonword repetition (NWR) tasks, the child is asked to repeat five words or nonwords five times to assess the variability of the speech of the child, which taps into the stages of motor planning and motor programming and stability of the phonological representation of the word form. The final task, maximum repetition rate (MRR), provides a window into the child’s motor execution by examining the child’s ability to repeat six different sequences as fast as possible (e.g., patakapataka…). For more information on the reliability, validation, and collection of the norms of the CAI, see van Haaften et al. [13].

### 2.4. Data Analysis

A computer or laptop with the CAI, which automatically stored the acoustic signal on the hard disk, was used. The children were seated in front of a microphone and wore open-back headphones to provide a good sound level of the automated instructions.

The recordings were transcribed (broad phonetic transcription) and analyzed according to the CAI examiner’s manual [37] on the computer by the student-SLPs or SLPs. The student-SLPs worked in pairs and the SLPs worked alone. Following the psychometric evaluation guidelines [13], all student SLPs and SLPs were required to practice the transcription and other analyses of the CAI with two practice-examples of children with SSD. After the training session, the results of the transcription and the analysis corresponded between the student-SLPs or SLPs. The transcriptions of the CAI of all children in this study were checked and differences were discussed between the student-SLPs or SLPs. The transcriptions were also checked by the first author (SD) or Anniek van Doornik (collaboration partner in collecting the data). After the transcription and analysis, an automated report was generated of several outcome measures of all CAI-tasks. The outcome measures (percentiles) were based on the data of the norm group [37]. Table 1 contains the outcome measures per speech task (parameters) used in the statistical analysis and the number of completed tasks per age group.

### 2.5. Statistical Analysis

All statistical analyses were performed using SPSS Version 26. All raw scores were transformed per age group (four/five/six years old) into z-scores to control for speech development to be able to compare the different variables with each other in a single analysis; the z-scores were calculated only with the raw scores of the 150 children with an SSD. To also control for outliers, z-scores lower than −2.33 or higher than 2.33 were replaced by −2.33 or 2.33, respectively; these were the lowest/highest z-scores observed in the CAI norm group. This was the case for eight z-scores in the entire database. Not all children could perform a correct sequence for the MRR task, due to speech–motor difficulties and/or due to shyness or inattentiveness of the child. Additionally, some recordings could not be analyzed due to the low acoustic quality. In cases where children made speech errors, for example replacing a sound with another sound, the missing score was replaced by the lowest z-score (−2.33) of the norm group. This was the case for ten children for the sequence pa; 15 for ta; 19 for ka; 59 for pataka; 29 for pata and 35 for taka.

A principal component analysis (PCA) with varimax rotation (listwise exclusion) was conducted to determine which components are present and to identify clusters of items.

The Kaiser-Meyer-Olkin (KMO) measure was calculated prior to the PCA to determine whether the sample size was adequate; a value larger than 0.5 is deemed acceptable [43]. The number of principals components (PC) was determined on the criterion for eigenvalues greater than 1. Components were retained if they featured at least three parameters. The CAI-parameters were considered for a PC if they had an absolute factor loading value of more than 0.4. The parameters with the highest factor loading on a PC were included in that PC [43].

Using the same procedure and criteria, a series of additional PCAs was performed subsequently on each of the subsets of variables loading significantly on one PC in the first analysis (see Table 2). There were several reasons to conduct this additional series. Because PCA necessarily applies listwise exclusion, the relatively large number of missing values in the MRR task also limited the number of datapoints for the other components. In the complementary PCAs per subset, all available data for that PC could be included. Factor loadings could thus be verified on all available data and composite performance scores could be obtained for the maximum number of children, including those with missing values on other PCs. These additional PCAs per subset also functioned as a check if the PCs should not be broken down into sub-components on the larger sample. Next, Pearson product-moment correlations were calculated to determine relationships between PCs. A split-half reliability of the PCs (comparing the outcomes when using half of the dataset, randomly selected, with the outcomes using the full dataset) was conducted to check whether the results were stable. If the results of the split-half procedure are similar to the results of the whole group this confirms the outcomes of the results of the conducted analysis.

Subsequently, we conducted an exploratory k-means cluster analysis with the z-scores of all CAI parameters to test whether distinctive profiles could be identified in our sample of children with SSD. K-means clustering is an unsupervised machine learning method to partition data into a predetermined *k* number of clusters. In an iterative manner, the observations are divided into groups in a way that minimizes the within-cluster variance and maximizes the variance between clusters. To determine which number of clusters provided the best fit, a comparison was made between analyses with two to four clusters. First, the Iteration History of every number of clusters was compared to determine the best solution. After this procedure, the graphs of the clusters were observed to see how the outcomes of the parameters were combined in the different clusters. For example, a two cluster-composition could mean the outcomes of the parameters are clustered in a group with children that score reasonably well and a group with children that score very low. Finally, the number of children in the different clusters was observed to see if there were clusters with a very small number of children in them.

In order to check for possible bias due to age or gender, the distributions of age and gender were compared across clusters. The construct validity was examined by comparing the clusters with respect to parameters of the CAI. The external validity (criterion) was also examined by comparing the clusters with the outcomes of the ICS (objective measure of severity), receptive vocabulary (PPVT-III-NL), auditory discrimination test (T-TOS), indication of the severity of the speech problem judged by the SLP and care givers (subjective measure of severity), the diagnosis given by the SLP and setting of the child (for example a private practice). This was analyzed with an ANOVA or a Chi-squared test, depending on the level of measurement of the variable; significance was defined as *p* < 0.05 for all tests.

## 3. Results

The results of the PCAs are presented first, along with the analysis of correlations between the PCs. Next, we describe the results of the cluster analysis, followed by a comparison between clusters of the PCs identified in the PCA as well as all the non-CAI variables. Note that all children in our sample have atypical speech development, which was verified with the percentage of consonants correct in syllable-initial position (PCCI) scores of the tasks Picture Naming and NonWord Imitation. The scores on these tasks were transformed into z-scores compared to the norm group. Note that these are different z-scores than used in the analysis of this study and were calculated with the average and the standard deviation of the norm group of the CAI [37]. All children scored below a z-score of −1.5 on at least one of the two parameters (PN-PCCI z-score *M* = −4.79, *SD* = 4.77; NWI-PCCI z-score M = −2.91, *SD* = 2.68), and no z-score higher than 1 occurred thus confirming the diagnosis of SSD for all children.

### 3.1. Principal Component Analysis

A PCA with orthogonal rotation (varimax) was conducted on all speech parameters of the CAI. The KMO measure confirmed adequacy of the sample for the analysis (KMO = 0.870). The analysis yielded a solution in which three components had an eigenvalue higher than 1, (12.7, 2.64 and 1.94 respectively). This three-component solution explained 61.7% of the variance. All principal components had a Cronbach’s alpha’s higher than 0.74, which indicates the internal consistency of the components were acceptable. The results of the PCA are presented in Table 2. Parameters loading high on the first PC were all the parameters of the PN task plus the following parameters of the NWI task: Level 5, Simplification processes and the Unusual processes (PN+) (an explanation of the parameters can be found in Table 1). The second PC included WR, NWR and almost all the parameters of the NWI task except for Level 5, Simplification processes and the Unusual processes (NWI/PWV). The last PC contained all the parameters of the MRR. It should be noted that the parameters NWI-PCCI, NWI-level 4, NWI-SP and NWI-UP also had high loadings (above 0.4) on one of the other two components; these parameters were included with the PC on which the highest loading was calculated. The grouping was confirmed by repeating the analysis with half of the SSD group.

A complementary series of PCAs was performed to obtain composite performance scores for all children, including those with missing values on other components, and to verify factor loadings and check if the PCs should not be broken down into sub-components on the larger sample. All three PCAs yielded a one-component solution. Within this additional PCA, the first component (PN+), comprising the PN parameters and the phonological processes of the NWI task explained 63.2% of the variance (KMO = 0.884); the second PC (NWI/PWV) comprising the remaining NWI parameters and the two repetition tasks (WR and NWR) explained 61.8% of the variance (KMO = 0.889), and the third PC (MRR) containing all MRR parameters explained 50.2% of the variance (KMO = 0.788). Pearson product-moment correlations between the components of the second PCA were calculated. Moderate and significant correlations were found between PN+ (PC 1) and MRR (PC 3), and between NWI/PWV (PC 2) and MRR (PC 3). The correlation between PN+ and NWI/PWV was high. The results are shown in Table 3.

### 3.2. Cluster Analysis

**Figure 2:**
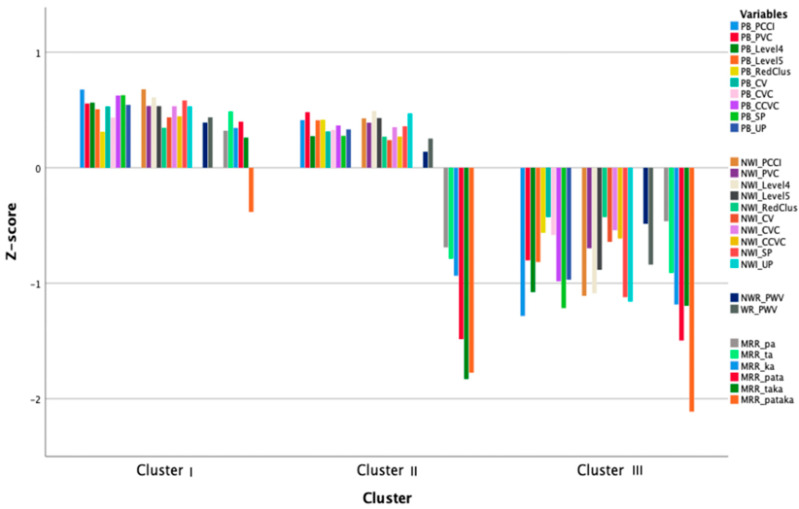
Overview of the distribution of the parameters across the clusters in z-scores.

A k-means cluster analysis was conducted with the same CAI parameters as used in the PCA (see Table 2). Forty-nine children out of a total 149 children were not included due to listwise exclusion (exclusion because of missing data); some children did not complete all the tasks due to failure or refusal. To check which number of clusters would fit best, the remaining 100 children were each allocated to one of either two, three or four clusters. The three-cluster analysis yielded the clearest results, which are shown in Table 4 and Figure 2. The two-cluster analysis yielded one group of children who performed poorly on all parameters and one group who performed slightly better on all parameters. The four-cluster analysis yielded one group that scored significantly worse and one group that performed significantly better, each compared to the other three clusters. However, no clear interpretation could be made of the profiles of the other two, intermediate clusters. Therefore, the analysis of three clusters was chosen to be described here. To validate this choice the same procedure was applied on a random selection of half of the 100 cases. The clusters yielded approximately the same mean scores for the three clusters as for the one based on all 100 children for the k-means cluster analysis, and the same components emerged for the PCA.

The three clusters that emerged differed significantly from each other with respect to the parameters PN-level 5, PN-CCVC, NWI-Level 4, NWI-Level 5, and all MRR parameters with all differences showing large effect sizes (*η*^2^ > 0.14). The children in cluster I outperformed children in cluster II and III, and children in cluster II scored better than children in cluster III. However, most of the CAI-parameters were not normally distributed, therefore, if a difference between the three groups was found to be significant at the 5% level, the comparison was reanalyzed using the Bonferroni corrected listwise comparisons for the non-normally distributed parameters. When this was applied, clusters I and II were not significantly different from each other on these parameters (Picture naming: PCCI, PVC, Level 4, RedClus, CV, CVC, SP, UP; NonWord Imitation: PCCI, PVC, RedClus, CV, CVC, SP, UP and the Word/NonWord Repetition), whereas cluster III was significantly different from clusters I and II. Children in cluster III scored lower than children in cluster I and II on all parameters. In Figure 2 the performance of the children on the tasks for the three clusters is shown.

### 3.3. Cluster Comparison with Non-CAI Variables

Two Chi-squared tests indicated that age and gender did not differ between the three clusters (see Table 4). A series of ANOVAs with post hoc pairwise comparisons indicated that the clusters did differ in the performance of the children on some of the additional assessments. With respect to the receptive vocabulary assessment (PPVT-III-NL), the auditory discrimination task (TTOS-ADT) and the speech intelligibility (ICS), the children in cluster I outperformed the children in clusters II and III while the cluster II children in turn also outperformed the children in cluster III (I > II > III; see Table 5).

The SLPs and caregivers were asked to rate the child’s speech problem in the questionnaire. SLPs and caregivers were asked ‘How would you estimate the severity of the speech problem?’ and they could answer with Mild, Moderate or Severe. To see if the SLP’s judgement correlated with the distribution in the clusters, a comparison (Chi-squared test) was made between the clusters with respect to the judgement of the severity of the SSD. There was a significant difference between the three clusters on the three severity levels; these differences showed a moderate effect size (*V* between 0.3 and 0.5) (see Table 5). Most of the children in cluster III were judged to have a severe speech problem, 19 (59.4%) children were considered to have a severe speech problem judged by SLPs and 13 (52.0%) children judged by their caregivers. The label moderate was mostly given to the children in cluster I and II. The label mild was given by the SLPs to 12 (80.0%) and by their caregivers to 16 (76.2%) children in cluster I; three children in cluster I were labeled by their caregivers as having no speech problem.

For 88 children, the diagnosis that they had received from their SLP was known (the diagnosis by the SLP based on the SLP’s assessment of the child); for 12 children, the SLP’s diagnosis was not known. The label phonological disorder was most often given by the SLPs followed by the diagnosis of CAS; phonetic disorder and dysarthria were the least frequent diagnoses. The result of the Chi-squared test showed no interaction between the diagnostic labels and the clusters.

The analysis also included the number of children attending a particular setting. The clusters differed significantly regarding setting. In clusters I and II, the largest category consisted of children who received speech therapy in a private practice. In cluster III, however, the largest category was special education for children with speech and language disorders. The settings audiologic center and rehabilitation center were divided roughly equally across the three clusters. The children who were initially recruited for the control group, but who turned out to have a speech problem, were mainly placed in Cluster I.

### 3.4. Comparison of Clusters and Components

To see if the clusters differed from each other on the scores on the three principal components (PC) identified in the PCA, a single multivariate ANOVA was conducted. The clusters differed significantly on each PC: PN+ (PC 1; *F* = 144.15, *p* = 0.000, *η*^2^ = 0.748); NWI/PWV (PC 2; *F* = 57.15, *p* = 0.000, *η*^2^ = 0.541) and MRR (PC 3; *F* = 88.66, *p* = 0.000, *η*^2^ = 0.646). Table 6 presents the mean PC scores for the three clusters. Children in the largest cluster (I, *n* = 46) scored best on all components. Children in cluster II (*n* = 28) showed a different pattern: they scored similar to children in cluster I on the PN+ PC and on the NWI/PWV, but they scored weak on the MRR. The children in cluster III (*n* = 26) scored very low on all the PCs.

## 4. Discussion

The overall aim of this study was to determine the possibility of profiling children with SSD based on underlying deficits. For this, the CAI was administered, and a two-step analysis procedure was conducted, comprising a Principal Component Analysis (PCA) to find components, followed by a cluster analysis (k-means clustering) to find distinct profiles. 

### 4.1. Step 1. Which Components Emerged and How Do These Compare to Norm Group Outcomes?

The PCA yielded three stable and meaningful components. The first component (labeled PN+) consisted of all picture naming (PN) parameters plus three parameters of the nonword imitation (NWI) task: NWI-level5 and the phonological processes (see Table 1 for explanation of the parameters). The second component (labeled NWI/PWV) consisted of the remaining NWI parameters and the two-proportion whole-word variability (PWV) parameters, based on word repetition (WR) and nonword repetition (NWR). The third component (labeled MRR) contained all maximum repetition rate (MRR) parameters. The results of the PCA in the current group of children with SSD differed from the results of the PCA in the CAI norm group, which consisted of Dutch children with typical development (*n* = 1.524) aged two to seven years [13]. In the norm group, five components were discovered: PN; segmental quality of NWI; quality of syllabic structure of NWI; word and nonword proportion of whole-word variability (PWV) and MRR. Note that the phonological processes were not included in the norm group, probably because their frequency of occurrence was too low among the 4–7-year-olds. The component MRR emerged as one component in both samples.

The five components from the norm group [13] were used in a previous study to compare the scores of 41 children with SSD [44]. That is, the components’ weights obtained from the PCA of the norm group were used to calculate component scores for the children with SSD. In this study, the child’s SLP had scored the severity of the speech disorder as moderate or severe (mild did not occur). Children in the moderate group obtained better scores than children in the severe group on parameters of the Picture Naming and NonWord Imitation tasks, whereas word and nonword repetition consistency were equal for these two groups. Furthermore, the moderate and severe groups differed with respect to the MRR-bi-and trisyllabic parameters, but not with respect to the MRR-monosyllabic sequences. Thus, this study provided evidence that comparison of performance on the different speech tasks of the CAI can provide distinct profiles which are different from the norm group and related to severity of SSD.

In the present study, not only the number of components in the clinical sample was smaller than that of the norm group, but also the composition of the first two components was different. In the norm group, all the parameters of the PN task loaded onto one component, and segmental quality of NWI and quality of syllabic structure of NWI on two separate components. In contrast, in the SSD group the specific phonological parameters of the NWI task, namely the NWI-level5, and the two phonological processes, loaded onto the PN component rather than on the NWI. Thus, the first component in the SSD group comprised both segmental and syllabic aspects of picture naming as well as specific phonological aspects of nonword imitation. Therefore, phonological encoding is a stronger component in the SSD group. The second component in the SSD group contained the remaining parameters of NWI, reflecting overall segmental quality (PCC, PVC) and quality of syllable structure (CV, CVC, CCVC), plus the percentage whole-word variability (WR-PWV and NWR-PWV). Interpretation? Related to the chain: auditory—memory—encoding/assembly. The difference between the norm group and the children with SSD regarding the parameters WR-PWV and NWR-PWV could be due to the fact that the typical children are consistent in this task already at an early developmental stage, resulting in a ceiling effect, whereas large differences were found between the SSD subgroups. Overall, the two components in the children with SSD as compared to four components in the norm group, seem to indicate a much clearer dissociation in the SSD group as compared to the norm group between phonological processes of speech production (word form retrieval and phonological encoding) and the processes that follow (motor planning, programming, and the stability of those processes). For naming pictures, children use the whole chain of the speech production process, and thereby rely on their vocabulary and –for the speech production process– specifically on the stored word forms (lexemes). In contrast, for repeating nonwords speakers use either the phonological decoding and encoding systems, or the auditory-to-motor-planning pathway (or both). The statistical result that PN and NWI-parameters load largely on different components, indicates that this distinction in underlying processing has significant impact on the quality of production. This implies that it is important to assess both tasks to get a broad view on the whole speech production process and on parts of the chain. Children who make relatively few errors in speech production when imitating nonwords may have relatively little difficulty in pronouncing new words they are learning, which could be a starting point for a method of intervention.

### 4.2. Which Clusters Emerged?

After the PCA analysis, a cluster analysis (k-means clustering) was conducted to see if subgroups would emerge from the data. Three clusters were found. The children in cluster I (*n* = 46) outperformed the children in the other two clusters on all parameters, while the children in cluster III (*n* = 26) scored lowest on all parameters. However, compared to the norm group, the children in cluster I scored lower on all parameters of PN and NWI. Although the cluster I group shows little or no vowel replacement in their speech as well as few errors in the simple syllable structures (CV and CVC), these children do make cluster reduction errors and phonological processes do still occur in more complex syllables. Therefore, this cluster can be labelled as phonological deficit. The children in cluster II (*n* = 28) showed a different pattern: they scored similar to the cluster I children on the PN+ and NWI/PWV principal components, but they scored weak on the MRR. As such, this cluster could be labeled as a phonological deficit with motoric deficit. The children in cluster III (*n* = 26) scored very low on all components, and this cluster could thus best be labeled as severe phonological and motoric deficit.

### 4.3. How Do the Different Clusters Compare to Each Other and to Norm Data?

McLeod [45] concluded in her review that 11 studies found a weak to moderately significant correlation between ICS and PCC. In our study this correlation calculation was not part of the research question, but we found a severity trend as well. As discussed above for each task of the CAI a difference can be observed between the clusters. This can be further supported by data on the intelligibility of the children as assessed by the caregivers and the SLPs. The intelligibility on the ICS is significantly different between the three clusters; the intelligibility of children in cluster I is better than that of cluster II and the children in cluster III show the lowest intelligibility. This was also confirmed by the responses of the speech therapists to the question of how severe they thought the speech problem was. Here, too, the clusters differed significantly from each other; the severity of the SSD is rated as least severe for the children in cluster I and more severe for the children in cluster II, and the children in 670 cluster III are the most severe cases according to the SLPs (severity: III > II > I).

With respect to error patterns, a first difference between the three clusters that can be observed is in vowel production. PN-PVC and NWI-PVC in cluster I and II showed a fairly high score and do not differ much from the children with a typical speech development. In typical development, five-year-old children achieve a mean PVC of 97.0 (SD = 3.9) in naming pictures and 90.5 (SD = 7.5) in repeating nonwords (see Table 3) [37]. The cluster I and II children in the present study showed similar averages and did not differ significantly from each other. However, the children in cluster III obtained significantly lower PVC-scores compared to the norm data. Roepke and Brosseau-Lapré [46] also observed differences in vowel production for 39 typically developing children compared to 45 children with SSD. They concluded that no conclusion could be drawn from their study as to whether these speech errors are systematic and reflect speech severity because the children were not matched on language ability but on age; another pattern might have been obtained if children were matched on language ability. However, a clear pattern was visible in our study: the children with the most severe speech disorder (cluster III; severe phonological and motoric deficit) showed lower PVCs than the other two less severe speech disorder groups.

Regarding consonant production, the results showed a similar profile among the clusters in the SSD group, cluster I and II children had similar averages of PCCI on both PN and NWI while the children in cluster III scored lower on both tasks. In the case of PCCI, however, all children with SSD scored lower compared to the norm group data (percentage for the five-year-old: PCCI-PN = 95.2, SD = 5.2; PCCI-NWI = 82.5, SD = 10.1). These findings indicate once more that measures such as the percentage consonants correct can serve as a severity index [47,48].

Consistency of errors was also measured in the present study, by means of the proportion whole-word variability when repeating five words and five nonwords five times (PWV-WR and PWV-NWR respectively). The children in cluster I and II scored the same and the children in cluster III were significantly less consistent in repeating the five words and nonwords. Compared to children in the norm group the mean inconsistency scores of the two tasks were slightly higher for the children in cluster I and II, and the children in cluster III showed the largest variability.

The last task of the CAI is the Maximum Repetition Rate (MRR). The results showed that children in cluster I outperformed children in cluster II and III on all MRR parameters and that the cluster II children outperformed the children in cluster III, all with a large effect size. In comparison to the norm group (mean of the five years old ranges from 3.74 syll/s to 4.29 syll/s for the different sequences in the norm group [37,49]), the children in cluster I scored similar on all MRR parameters. The children in cluster II produced the monosyllabic sequences slightly slower than the children of the norm group, and the bi- and trisyllabic sequences were produced at least one syllable per second slower than the norm group. The cluster III children produced the /pa/ sequences somewhat slower than the norm group as well and produced all other sequences with at least one syllable per second slower [37,49]. Children in cluster II and III were slightly better on the mono syllabic sequences compared to the bi-tri syllabic sequences. This difference may be a predictor of motor planning and programming problems. Ozanne [50] performed a cluster analysis of 18 behaviors that could reveal an underlying speech motor planning and programming problem on a dataset in a study of 100 children (ages 3;0–5;6 years;months) with SSD of unknown origin. The most common problems of the children were incorrect DDK sequences (38%), slow DDK rate (35%) and an increase in errors with increased linguistic load (27%), which corroborates our findings.

In the past, several debates have taken place about the potential value of nonspeech oral motor tasks such as the MRR [51]. Criticism has mainly come from the field of adult acquired disorders, but most studies with children conclude that MRR should be part of the assessment of SSD [52,53,54]. The current study confirms that MRR performance has a distinctive contribution to the diagnosis of SSD. The distinction between Cluster I and II is primarily based on MRR, and the distinction between Cluster I and III on both MRR and the phonological components. Across clusters, the correlations between the phonological components are high, and the correlations between these clusters and MRR are moderate. This shows that MRR contributes to diagnostic classification as an indicator of speech motor involvement (Cluster 1 versus Cluster 2) and can be considered an indicator of severity (Clusters 2 and 3). 

In summary, three conclusions can be drawn from the analysis of the clusters: (1) there are different profiles of SSD; (2) in which severity plays a role and (3) that cover a spectrum of degrees of involvement of different underlying problems.

In this study, the group of children with missing values in the MRR, because children could not or refused to perform a sequence, was not included in the cluster analysis. Why the children refused is not known; children were not asked to give an explanation. They might have refused out of boredom of the session. In addition, not all typically developing children in the MRR norm group performed a sequence either [48]. In the future, qualitative analysis (e.g., 0 = no MRR; 1 = could not perform a long sequence; 2 = could not perform a sequence correctly due to a speech error, etc.) could be used to assess the number of children who performed a sequence.

### 4.4. How Do These Relate to Diagnostic Classification Systems?

We cannot make a direct, quantitative comparison, between our results and the results of the previously mentioned two studies in the introduction classifying children with the SCDC [29] and with Dodd’s model [28], due to the large differences in tasks and data analysis method. This study applied a data-driven cluster analysis, while the other two studies aimed to classify the children according to pre-determined profiles that (are assumed to) correspond to certain subtypes of speech disorders. Furthermore, in our data, severity of the speech disorder also plays a role in clustering the outcomes of the CAI, while speech severity is not included in the validity studies of SCDC and in Dodd’s model.

#### 4.4.1. Dodd’s Model for Differential Diagnosis (MDD)

The children in the consistent atypical phonological disorder group and the children in the phonological delay group in the study of Ttofari-Eecen et al. [28] had at least one (a)typical phonological error pattern and had no difficulty repeating the 25 words of the DEAP Inconsistency Assessment [32] multiple times. This group could be compared to the children in cluster I, who also had at least one typical and/or atypical phonological error pattern. However, children in cluster I had a higher mean score on the Word and NonWord repetition tasks of the CAI compared to the norm group of the CAI; children in cluster I scored les consistent. Therefore, they might not be similar to the consistent atypical phonological disorder group and the phonological delay group of the MDD model; these children do not have a lower score on an inconsistency assessment compared to a norm group. The children in cluster II performed similar to the children in the inconsistent phonological disorder group of the Ttofari-Eecen-study; they had a typical and/or atypical phonological error pattern and were inconsistent in their speech. The children in cluster III can be compared with the suspected atypical speech motor control group based on the overall low scores on the CAI-parameters, including the MRR-task. Ttofari-Eecen et al. [28] also found oromotor problems in their population; unfortunately, the results of the Dutch oromotor task was not known for all children in our study.

#### 4.4.2. Speech Disorders Classification System (SDCS) 

Comparison with the SDCS is even more complicated as the different categories of the SDCS are defined at different levels: etiological processes (distal causes), speech processes (proximal causes), and clinical typology (behavioral phenotype) [55]. Focusing on the categories based on clinical typology, the children in cluster I (phonological deficit cluster) can probably best be compared to children in the Speech Delay group (SD), as they showed no evidence of motor involvement (scores on PWV and MRR that are only slightly below the norm). The children in the other two clusters, with poor MRR would probably fall within the Motor Speech Disorder group (MSD). Further differentiation between subgroups of MSD requires additional speech motor tasks, which is beyond the scope of this study.

### 4.5. Clinical Implications and Future Research

In the future, the tasks of the CAI will be supplemented with components that can provide a more detailed view of problems with motor planning and programming. Examples of these components: are systematic manipulation of conditions during speech such as speeding up; blocking auditory feedback and exercises to determine a short-term learning effect [9]; as well as acoustic measurements of coarticulation and variability [56]. The aim of the CAI is to provide SLPs with sufficient information to plan a well-fitting intervention that is specifically tailored to the individual child. In 2010, Williams et al. reported 23 different interventions for children with SSD [57]. There are currently even more interventions available that were not included in that article, for example the Dynamic Temporal and Tactile Cueing (DTTC) [58] and since 2010 a few new interventions entered the market, for example Rapid Syllable Transition Treatment (ReST) [59]. More fine-grained analyses of underlying processing deficits could give a large contribution to the design of tailor-made therapy plans.

A classification of the different interventions and mapping these onto the outcomes of the process-oriented assessment might be a solution, as already described by several authors [12,60,61]. In their review, Wren and colleagues [61] proposed a framework of five different categories of interventions: (1) environmental, (2) auditory–perceptual, (3) cognitive–linguistic, (4) production, and (5) integrated. For the children in the present study, it would perhaps be best to offer the children in cluster I (phonological deficit cluster) an intervention in the auditory-perceptual category or the cognitive-linguistics category because as the results show these children showed problems mainly with the tasks PN and NWI. This suggests that these children experience problems primarily in lemma access, word form retrieval, and phonological encoding. To treat these problems, the SLP can choose an intervention that falls under the auditory perceptual interventions or the cognitive-linguistic interventions. The auditory perceptual interventions target the perceptual skills of the child to change the speech output. The aim is to immerse the child in an auditory stimulation of word targets as well as auditory discrimination exercises that stimulate the child’s phonemic awareness, for example cycles approach. The cognitive-linguistics interventions stimulate the higher-level processing to promote change in the speech through confronting a child with their reduced set of contrasts or increasing awareness of sounds in speech, for example Metaphon [61]. To help SLPs make a choice between these two interventions, Bron et al. [62] developed a flowchart for Dutch SLPs in which, for example, age is one of the factors. Younger children could have more difficulty with a cognitive-linguistic intervention (such as Metaphon), because this form of intervention relies more on the child’s cognitive abilities; the children must learn to hear the differences between their pronunciation of the word and the correct pronunciation and they must also understand that their pronunciation refers to a different concept than the word they mean to pronounce, for example the difference between ‘hat’ and ‘rat’. Younger children or children with lower cognitive abilities and/or children with an inconsistent error pattern tend to benefit more from a phonological cycles approach then from Metaphon [62].

The second group of children (cluster II, phonological and motoric deficit) scored worse on the speech motor tasks of the CAI (sequences of the MRR with more problems with the bi-tri syllabic sequences) than the children in cluster I; they also have more problems with the pronunciation of /l/ and /r/ (level 5) and the CCVC structures. These children have problems with the following underlying speech processes: lemma access, word form selection, phonological encoding and speech motor planning and programming (see Figure 1). The interventions in the category production could be a good choice; they can benefit from the guidance on phonetic placement or manner and imitation in combination with one of the interventions in the auditory perceptual or the cognitive-linguistic group.

The last group of children (cluster III, severe phonological and motoric deficit) score low on all the tasks and especially on the speech motor tasks. What also distinguishes this group from the other two clusters is the additional lower score on the auditory discrimination task. Integrating an auditory perceptual intervention with one that is more focused on the motor speech system (production) could help to fill the child’s phonological system and reduce the speech motor difficulties. Currently, SLPs combine interventions and usually choose the intervention based on availability and own experience [18,19]. Hopefully this will change in the future and SLPs will make their decisions during clinical reasoning on a process-oriented assessment and the framework described by Wren et al. [61]. Further development of treatment planning frameworks, flow charts and decision trees on additional assessments leading to specific treatment recommendation/prescription are warranted.

## 5. Conclusions

In summary, the results of this study demonstrated three underlying principal components of the CAI-parameters for a group of children with SSD. The components showed a different pattern compared to a study with typically developing children with the same CAI-parameters. Three different clusters of children could be identified. The largest group showed problems compared to the norm group only at the phonological level and could be characterized as having a phonological deficit. The second, much smaller group had the same problems but also experienced some difficulties at the speech motor level. This group was termed as having phonological and motor deficits. The third group, equal in number to the second, showed extensive problems at both the phonological and speech motor level and could be characterized as having severe phonological and motor deficits. This data-driven clustering shows that there seems to be a difference in severity of the speech disorders amongst the three clusters, and different profiles of speech processing problems could be detected in our sample. The profiles are informative with respect to treatment planning in that each profile implies a specific intervention approach. More comparative research is needed to test the diagnostic accuracy of process-orientated diagnosis methods including more and different children, for example children with dysarthria, and controlling for possible additional factors such as behavioral characteristics and language impairment [63].

## Figures and Tables

**Table 1:** Parameters/outcome measures per speech task.

Task	Parameter		*n*
PN	PCCI	Percentage of consonants correct in syllable-initial position	149
	PVC	Percentage of vowels correct	149
	Level 4	Percentage of correct consonants /b/, /f/ and /ʋ/	149
	Level 5	Percentage of correct consonants /l/ and /R/	149
	RedClus	Percentage of reduction of initial consonant clusters from 2 consonants to 1	149
	CV	Percentage of correct syllable structure CV	149
	CVC	Percentage of correct syllable structure CVC	149
	CCVC	Percentage of correct syllable structure CCVC (C = consonant, V = vowel)	149
	SP	Simplification processes, total score of the processes: fronting, stopping of fricatives, voicing, devoicing and gliding	149
	UP	Unusual processes, total score of the processes: backing, unusual stopping, Hsation, nasalisation and denasalisation	149
NWI	PCCI	Percentage of consonants correct in syllable-initial position	146
	PVC	Percentage of vowels correct	146
	Level 4	Percentage of correct consonants /b/, /f/ and /ʋ/	146
	Level 5	Percentage of correct consonants /l/ and /R/	146
	RedClus	Percentage of reduction of initial consonant clusters from 2 consonants to 1	146
	CV	Percentage of correct syllable structure CV	146
	CVC	Percentage of correct syllable structure CVC	146
	CCVC	Percentage of correct syllable structure CCVC	146
	SP	Simplification processes, total score of the processes: fronting, stopping of fricatives, voicing, devoicing and gliding	146
	UP	Unusual processes, total score of the processes: backing, unusual stopping, Hsation, nasalisation and denasalisation	146
WR	PWV	Proportion of whole-word variability—Word repetition	149
NWR	PWV	Proportion of whole-word variability—Nonword repetition	147
MRR	pa	Number of syllables per second of sequence /pa/	133
	ta	Number of syllables per second of sequence /ta/	133
	ka	Number of syllables per second of sequence /ka/	131
	pata	Number of syllables per second of sequence /pata/	120
	taka	Number of syllables per second of sequence /taka/	115
	pataka	Number of syllables per second of sequence /pataka/	111

Note. PN = Picture Naming; NWI = NonWord Imitation; WR = Word Repetition; NWR = NonWord Repetition; MRR = Maximum Repetition Rate. Note. Level 4 and 5 are part of the five degrees of complexity of phonological contrasts of Dutch syllable-initial consonants described by Beers [42].

**Table 2:** Principal Component Analysis results for Picture Naming (PN), NonWord Imitation (NWI), Word (WR) and NonWord (NWR) Repetition, and Maximum Repetition Rate (MRR). The highest component loading of each parameter is displayed in boldface.

Task	Parameter	Component		
		1	2	3
PN	PCCI	** 0.896 **	0.262	0.224
	PVC	** 0.655 **	0.430	0.177
	RedClus	** 0.817 **	0.089	0.163
	Level 4	** 0.728 **	0.170	0.180
	Level 5	** 0.631 **	0.237	−0.053
	CV	** 0.432 **	0.249	0.400
	CVC	** 0.563 **	0.364	0.080
	CCVC	** 0.797 **	0.262	0.176
	SP	** 0.801 **	0.197	0.185
	UP	** 0.768 **	0.199	0.150
NWI	PCCI	0.601	** 0.730 **	0.153
	PVC	0.367	** 0.823 **	0.155
	RedClus	0.561	** 0.648 **	0.072
	Level 4	0.508	** 0.680 **	0.053
	Level 5	** 0.469 **	0.319	0.124
	CV	0.104	** 0.731 **	0.283
	CVC	0.349	** 0.715 **	0.257
	CCVC	0.477	** 0.481 **	0.050
	SP	** 0.632 **	0.532	0.133
	UP	** 0.637 **	0.542	0.002
WR	PWV	0.085	** 0.730 **	0.111
NWR	PWV	0.284	** 0.566 **	0.175
MRR	pa	−0.202	0.271	** 0.726 **
	ta	−0.004	0.240	** 0.786 **
	ka	0.202	0.094	** 0.667 **
	pata	0.230	0.130	** 0.708 **
	taka	0.198	−0.148	** 0.720 **
	pataka	0.255	0.226	** 0.445 **
Eigenvalues		12.70	2.64	1.94
% of variance		45.37	9.42	6.93
Cronbach’s α		0.945	0.909	0.796

Note. PN = Picture Naming; NWI = NonWord Imitation; WR = Word Repetition; NWR = NonWord Repetition; MRR = Maximum Repetition Rate; PCCI = Percentage of consonants correct in syllable-initial position; PVC = Percentage of vowels correct; Level 4 = percentage of correct consonants /b/, /f/ and /ʋ/; Level 5 = percentage of correct consonants /l/ and /R/; RedClus = percentage of reduction of initial consonant clusters from 2 consonants to 1; CV = percentage of correct syllable structure CV; CVC = percentage of correct syllable structure CVC; CCVC = percentage of correct syllable structure CCVC; SP = Simplification processes, total score of the processes: fronting, stopping, voicing, devoicing and gliding; UP = Unusual processes, total score of the processes: backing, atypical stopping, Hsation, nasalisation and denasalisation; WR-PWV = Proportion of whole-word variability—Word Repetition; NWR-PWV = Proportion of whole-word variability—NonWord Repetition; MRR-pa = number of syllables per second of sequence /pa/; MRR-ta = number of syllables per second of sequence /ta/; MRR-ka = number of syllables per second of sequence /ka/; MRR-pataka = number of syllables per second of sequence /pataka/; MRR-pata = number of syllables per second of sequence /pata/; MRR-taka = number of syllables per second of sequence /taka/.

**Table 3:** Pearson correlations between factors, *n* = 100.

Factors	PN+	NWI/PWV	MRR
PN+	-	0.793 *	0.420 *
NWI-/PWV	0.793 *	-	0.375 *
MRR	0.375 *	0.420 *	-

Note. PN = Picture Naming; NWI = NonWord Imitation; PWV = proportion of whole-word variability, Word and NonWord Repetition; MRR = Maximum Repetition Rate. * Correlation of factor scores is significant at the 0.01 level (two-tailed).

**Table 4:** Measures age, gender, parameters of the CAI in three subgroups of children with SSD identified by cluster analysis (*n* = 100).

Variable		Norm Group	Cluster			ANOVA		
		(*n* = 121)	I (*n* = 46)	II (*n* = 28)	III (*n* = 26)	*F*	*p*	*η* ^2^
Age (age in months (SD))		61.5 (1.10)	62.1 (8.40)	60.2 (9.09)	61.3 (8.69)	0.404	0.669	0.008
*n* and (%) boys		66 (54.5%)	28 (49.1%)	18 (31.6%)	11 (19.3%)	3.177	0.204	0.178
PN	PCCI	96.8 (3.7)	90.6 (6.99)	85.0 (11.25)	56.2 (12.31)	106.197	<0.001 * ^I = II, I/II > III^	0.686
	PVC	97.7 (3.1)	97.7 (2.97)	97.0 (2.59)	87.0 (7.81)	48.267	<0.001 * ^I = II, I/II > III^	0.499
	Level 4	~	90.5 (13.53)	82.1 (20.89)	46.6 (28.06)	40.478	<0.001 * ^I = II, I/II > III^	0.455
	Level 5	93.6 (11.0)	73.1 (20.87)	69.8 (20.96)	34.7 (24.69)	27.78	<0.001 *	0.364
	RedClus	97.0 (6.2)	89.5 (16.05)	89.0 (14.63)	69.3 (23.25)	12.080	<0.001 * ^I = II, I/II > III^	0.199
	CV	~	94.9 (6.09)	91.5 (10.12)	78.5 (17.22)	18.864	<0.001 * ^I = II, I/II > III^	0.280
	CVC	~	93.9 (5.12)	92.3 (6.89)	82.6 (10.30)	21.553	<0.001 * ^I = II, I/II > III^	0.308
	CCVC	94.4 (9.9)	82.1 (20.14)	70.7 (25.20)	30.4 (22.81)	45.436	<0.001 *	0.484
	SP	2.8 (5.2)	13.4 (13.26)	27.9 (27.44)	82.8 (38.47)	61.197	<0.001 * ^I = II, I/II > III^	0.558
	UP	0.2 (0.5)	2.8 (4.16)	5.5 (7.28)	19.6 (11.68)	42.634	<0.001 * ^I = II, I/II > III^	0.468
NWI	PCCI	87.7 (6.9)	78.3 (13.30)	71.9 (15.61)	39.4 (9.99)	74.758	<0.001 * ^I = II, I/II > III^	0.607
	PVC	93.5 (4.8)	91.9 (8.41)	88.6 (11.29)	70.5 (14.96)	31.844	<0.001 * ^I = II, I/II > III^	0.396
	Level 4	87.8 (11.8)	76.3 (18.63)	72.5 (20.23)	30.3 (18.26)	53.376	<0.001 *	0.524
	Level 5	87.2 (12.8)	72.0 (20.70)	68.8 (25.10)	31.1 (17.74)	33.562	<0.001 *	0.409
	RedClus	92.3 (11.8)	86.6 (14.94)	85.2 (18.81)	68.4 (23.37)	8.843	<0.001 * ^I = II, I/II > III^	0.154
	CV	96.8 (7.8)	94.8 (9.04)	92.9 (13.15)	74.4 (21.14)	18.770	<0.001 * ^I = II, I/II > III^	0.279
	CVC	93.3 (5.7)	90.96 (8.18)	87.32 (9.15)	71.5 (18.12)	23.551	<0.001 * ^I = II, I/II > III^	0.327
	CCVC	83.0 (25.2)	75.1 (30.40)	67.9 (36.88)	32.1 (35.98)	14.043	<0.001 *	0.225
	SP	7.2 (7.4)	27.5 (23.86)	39.8 (30.29)	105.2 (43.27)	52.502	<0.001 * ^I = II, I/II > III^	0.520
	UP	2.1 (2.2)	9.7 (7.20)	11.9 (8.65)	34.1 (14.24)	55.043	<0.001 * ^I = II, I/II > III^	0.532
WR	PWV	0.23 (0.04)	0.30 (0.07)	0.32 (0.11)	0.47 (0.16)	21.483	<0.001 * ^I = II, I/II > III^	0.307
NWR	PWV	0.28 (0.08)	0.35 (0.12)	0.40 (0.15)	0.51 (0.21)	9.242	<0.001 * ^I = II, I/II > III^	0.160
MRR	pa	4.64 (0.61)	4.44 (0.51)	3.82 (0.58)	3.97 (0.78)	7.092	0.001 *	0.143
	ta	4.44 (0.60)	4.45 (0.44)	3.65 (0.68)	3.53 (0.83)	17.092	<0.001 *	0.284
	ka	4.34 (0.51)	4.01 (0.59)	3.14 (0.80)	2.91 (0.91)	11.255	<0.001 *	0.220
	pata	4.49 (0.73)	4.59 (0.92)	2.81 (0.88)	2.78 (1.01)	23.710	<0.001 *	0.404
	taka	4.37 (0.75)	4.32 (0.79)	2.64 (0.56)	3.15 (0.99)	23.332	<0.001 *	0.418
	pataka	4.09 (0.82)	3.47 (1.18)	2.33 (0.57)	2.81 (1.07)	12.745	<0.001 *	0.372

Note. PN = Picture naming; NWI = Nonword imitation; WR = Word repetition; NWR = Nonword repetition; MRR = Maximum repetition rate; PCCI = Percentage of consonants correct in syllable-initial position; PVC = Percentage of vowels correct; Level 4 = percentage of correct consonants /b/, /f/ and /ʋ/; Level 5 = percentage of correct consonants /l/ and /R/; RedClus = percentage of reduction of initial consonant clusters from 2 consonants to 1; CV = percentage of correct syllable structure CV; CVC = percentage of correct syllable structure CVC; CCVC = percentage of correct syllable structure CCVC; SP = Simplification processes, total score of the processes: fronting, stopping, voicing, devoicing and gliding; UP = Unusual processes, total score of the processes: backing, atypical stopping, Hsation, nasalisation and denasalisation; WR-PWV = Proportion of whole-word variability—Word repetition; NWR-PWV = Proportion of whole-word variability—Nonword repetition; MRR-pa = number of syllables per second of sequence /pa/; MRR-ta = number of syllables per second of sequence /ta/; MRR-ka = number of syllables per second of sequence /ka/; MRR-pataka = number of syllables per second of sequence /pataka/; MRR-pata = number of syllables per second of sequence /pata/; MRR-taka = number of syllables per second of sequence /taka/, ~ no score because of a ceiling effect in the norm group. Note. Redclus for the norm group is inverted. Note. SP and UP: a lower score means better performance. Note. * ANOVA is significant at the 0.01 level. Note. The coding below the *p*-value are the results of the post-hoc analysis, e.g., ^I = II, I/II > III^ means: differences between cluster I and II are not significant, whereas I and II outperform III.

**Table 5:** Measures PPVT-III-NL, TTOS-ADT, ICS, intelligibility level SLP and parents, diagnosis and setting in three subgroups of children with SSD identified by cluster analysis (*n* = 100).

Variable		Cluster			ANOVA for Continuous and χ^2^ for Categorical Variables		η^2^ for Continuous and V for Categorical Variables
		I (*n* = 46)	II (*n* = 28)	III (*n* = 26)			
PPVT-III-NL		102.8 (14.00) ^$^	101.5 (10.94) ^$$^	90.8 (11.86) ^$$$^	5.201	0.008 *	0.152
T-TOS (ADT)		63.4 (27.67) ^+^	52.9 (34.58) ^++^	34.4 (25.17) ^+++^	5.959	0.004 *	0.153
ICS		4.0 (0.40) ^^^	3.8 (0.44) ^^^^	3.5 (0.51) ^^^^^	9.801	<0.001 *	0.201
Intelligibility affected (SLPs) (*n* = 73)					28.027	<0.001*	0.438
	mild	12 (80.0%)	3 (20.0%)	0 (0.0%)			
	moderate	11 (42.3%)	11 (42.3%)	4 (15.4%)			
	severe	5 (15.6%)	8 (25.0%)	19 (59.4%)			
Intelligibility level (parents) (*n* = 78)					22.478	0.001 *	0.380
	no speech problem	3 (100.0%)	0 (0.0%)	0 (0.0%)			
	mild	16 (76.2%)	3 (14.3%)	2 (9.5%)			
	moderate	10 (34.5%)	11 (37.9%)	8 (27.6%)			
	severe	5 (20.0%)	7 (28.0%)	13 (52.0%)			
Diagnosis (*n* = 88)					7.266	0.297	0.058
	Phonetic disorder	5 (62.5%)	2 (25.0%)	1 (12.5%)			
	Phonological disorder	27 (42.2%)	17 (26.6%)	20 (31.3%)			
	Childhood Apraxia of Speech (CAS)	3 (25.0%)	6 (50.0%)	3 (25.0%)			
	Dysarthria	0 (0.0%)	2 (50.0%)	2 (50.0%)			
Setting					32.744	<0.001 *	0.405
	Private practice	22 (53.7%)	15 (36.6%)	4 (9.8%)			
	Special education	9 (26.5%)	6 (17.6%)	19 (55.9%)			
	Rehabilitation centre	4 (33.3%)	5 (41.7%)	3 (25.0%)			
	Audiologic centre	1 (50.0%)	1 (50.0%)	0 (0.0%)			
	Recruited as control group	10 (90.9%)	1 (9.1%)	0 (0.0%)			

Note. PPVT-III-NL = quotient of word comprehension; TTOS-ADT = percentile of auditory discrimination test. ICS = average score on the Intelligibility of Context Scale. Note. Data missing in this group: ^$^ 16 children, ^$$^ 15 children, ^$$$^ 8 children; ^+^ 13 children; ^++^ 11 children, ^+++^ 7 children; ^^^ 13 children, ^^^^ 5 children, ^^^^^ 1 child Note. Diagnosis: some children did not get a diagnosis, because the SLP did not include it in the questionnaire or the child was part of the control group. Note. * ANOVA is significant at the 0.01 level.

**Table 6:** Mean factor scores and standard deviation of the three clusters per factor.

	Factor					
	PN+		NWI-/PWV		MRR	
Cluster	M	SD	M	SD	M	SD
I	0.72	0.46	0.64	0.61	0.87	0.58
II	0.49	0.50	0.41	0.69	−0.76	0.46
III	−1.25	0.52	−0.96	0.58	−0.72	0.77

## Data Availability

The data presented in this study are available on request from the corresponding author. The data are not publicly available due to articles in preparation.
